# Defining a universal measurement unit and scale for gross motor development

**DOI:** 10.3389/fresc.2024.1243336

**Published:** 2024-01-19

**Authors:** Bryant A. Seamon, Cynthia L. Sears, Emily Anderson, Craig A. Velozo

**Affiliations:** ^1^ College of Health Professions, Department of Rehabilitation Sciences, Division of Physical Therapy, Medical University of South Carolina, Charleston, SC, United States; ^2^ College of Health and Society, Occupational Therapy Program, Hawai’i Pacific University, Honolulu, HI, United States; ^3^ Augusta Therapy Services for Children, LLC, North Augusta, SC, United States; ^4^ College of Health Professions, Department of Rehabilitation Sciences, Division of Occupational Therapy, Medical University of South Carolina, Charleston, SC, United States

**Keywords:** measures, pediatrics, growth, child development, motor skills

## Abstract

**Introduction:**

The ability of children to accomplish progressively more difficult gross motor tasks follows a predictable sequence that has been well documented as part of development. Current existing instruments were developed independently using classical test theory methods which led to the lack of a universal measurement scale and unit. The purpose of this study was to test a specification equation, anchored to commonly accepted and reproducible tasks in gross motor development, to generate a universal measurement scale and unit of measurement, called the Gross Motor (GM) unit.

**Methods:**

We rated component measures for each of the gross motor development tasks on the Gross Motor Function Measure-66 (GMFM). The GMFM is a gross motor development measure created with Rasch measurement theory to quantify observed difficulty levels measured on an interval scale. Component measures for body position, movement, and support were based on hypothesized contributions to gross motor development based on theory. Forward stepwise linear regression was used to test a specification equation. The specification equation was anchored to reference points to fix a unit size.

**Results:**

Our specification equation explained 87% of the variance in observed gross motor task difficulty. Predicted difficulty for gross motor tasks was strongly associated with observed task difficulty (*r* = 0.94, *p* < 0.0001). Our specification equation was anchored to 1) lying supine (0 GM units) and 2) walking unsupported (100 GM units) setting the size of the GM unit to 1/100 of the distance between lying supine and unsupported walking.

**Discussion:**

Our specification equation allows for experimental testing of gross motor development theories. This approach provides a framework for refining our understanding and measurement of gross motor development and creates a universal scale and unit. We expect that this will facilitate placing many, if not all, current gross motor development instruments on the same measurement scale.

## Introduction

The progression of gross motor development during childhood is well known, in part, because it is an observable, global human experience. Gross motor development typically occurs through predictable sequencing and patterns including proximal to distal stability, cephalic to caudal progression of movement against gravity, movement of the body in stationary positions which allows for the development of balance and coordination skills, and finally manipulation of objects within the environment for further exploration (1). Identifying a child's location along the development progression is important because early detection and prevention of gross motor delays have proven to be effective and efficient in maximizing physical health (2). Measurement of gross motor development allows for quantifying a child's location and is imperative to identify any delays. Currently, there are more than 200 pediatric assessments or instruments used to measure a child's gross motor development (3–5). There are several challenges that can arise from having multiple measurement instruments (6, 7) but the greatest is arguably the inability to easily equate measures across tools for meaningful decision making (8, 9).

Measures from existing instruments cannot be equated because of two critical problems; (1) they have been developed from data rather than theory, and (2) they lack a universal measurement unit that is standardized and reproducible (10–14). Developing instruments from data sets with group-level analytical approaches will always cause measures to be explicably tied to the sample studied (i.e., sample dependent) (15). Sample dependent measures are very common in social and behavioral sciences because of classical test theory methodology for instrument development (10, 12). Classical test theory places an emphasis on the development of test items that describe an underlying construct and analyzing the items’ performance collectively by examining the relationship between total scores and other metrics across groups to establish validity (15). This methodology causes scores or tests to be the focus of measurement rather than the underlying constructs that contribute to the observable phenomenon of interest (10). The contrasting approach would be to develop measures from mathematical models built from testing underlying constructs informed by an overall theory for explaining the phenomenon of interest (13, 16–19).

The physical sciences provide an ideal example of this theory-driven approach for measurement development (10). An example of this approach is how temperature is measured. Physicists initially observed temperature by the expansion of liquid in glass tubes (20). This then led to a series of experiments by Daniel Fahrenheit that showed temperature manipulation yielded a reliable change in mercury's expansion (21). The amount of chemical expansion could then be mathematically modeled to derive a formula, or specification equation, for quantifying temperature (21). This methodological approach demonstrates how construct theory for an observed phenomenon (i.e., a chemical's expansion is explained by temperature) can be exposed to falsification. Karl Popper's Falsification Principle states that a theory is falsifiable or refutable if it can be disproven by experiments and empirical evidence (22). Falsification can be accomplished by testing the accuracy of a specification equation to predict changes in the construct. When contributing components to a specification equation are not able to accurately predict changes in the construct then the theory underlying the specification equation has been falsified or disproved. Daniel Fahrenheit demonstrated this by testing whether mercury expanded by the mathematically predicted amount when exposed to a specific temperature (21). The ability to expose theory to falsification provides a better understanding of the construct theory and a clear path for how to improve the instrument and theory (16).

Another critical problem with measures for gross motor development is the lack of a universal measurement unit (10, 11, 16, 17). Measurement units are defined as a “scalar quantity, defined and adopted by convention, with which any other quantity of the same kind can be compared to express the ratio of the two quantities as a number” (23). Existing instruments are reliant on total scores that are not composed of linear or interval unit which has led to measurement scales that are independent from one another without comparable quantities (15). As a result, meaning of measurement values must be derived through relation to normative data (e.g., T-scores), predictive ability, or showing differences across groups (e.g., normal children vs. individuals with disability) rather than through a comparable unit connected to theory (10). The emergence of item response theory and Rasch measurement theory has been able to create linear and interval measurement units for many existing scales (12). However, the measurement scales derived from item response theory models are limited to local objectivity and measurement units are not analogous because of sample dependency (10). The physical sciences have been able to achieve general objectivity through imposing anchors and standardized unit sizes (10, 13). For example, the Celsius measurement scale is anchored to the temperature that water freezes (0°C) and boils (100°C) with 1°C (i.e., unit size) equal to 1/100 (20). Stenner and colleagues (10, 16, 17) have argued that anchoring measures provides general objectivity because the unit size and scale are based on theory rather than observations from a specific sample of people (i.e., local objectivity). Anchor points that are reproducible and widely recognized provide valuable reference points for interpreting measurement values across the scale (10). They also allow for consolidating instruments by placing them on the same scale (10, 11).

Given the proliferation of gross motor development measures and critical concerns related to instrument development there is an explicit need to develop an explanatory model based on theory and create a universal measurement unit of gross motor development. An explanatory model will facilitate exposing the theory of gross motor development to falsification through the creation and testing of a specification equation (10, 13, 16). Additionally, developing a universal measurement unit and imposing general objectivity will equate existing measures to a standardized scale and provide meaning to units that is reflective of gross motor development theory (10, 11). The best example of this measurement approach being applied in the social and behavioral sciences is the Lexile measurement scale and unit (Lexile; L) for reading ability and passage difficulty developed by Stenner (10). The Lexile was developed from theory by testing the relationship between reading difficulty and various hypothesized variables thought to contribute to how challenging a passage of text is to comprehend. The Rasch measurement model calibrated passage difficulty and an individual's reading ability onto a linear, interval scale allowing the research team to quantify the phenomenon of reading comprehension. Stenner then used linear regression to derive a specification equation for testing the relationship between hypothesized variables explaining passage difficulty with actual passage difficulty (10). This process exposed their theory to falsification by testing which hypothesized variables accounted for the most variance in passage difficulty (10). Stenner demonstrated that a semantic component (mean log of word frequencies in a passage) and syntactic component (log mean sentence length in a passage) were all that was required to explain the variance in passage difficulty and, by extension, persons reading ability (*R*^2 ^= 0.85) (10). Stenner then established general objectivity through a universal unit of reading ability by algebraically anchoring the specification equation to derive Lexiles. The specification equation was anchored to the difficulty of a set of basal primer texts (200 Lexiles) and encyclopedia texts (1,200 Lexiles) with one Lexile equal to 1/1,000 of the distance between texts (10). The Lexile measurement scale has since equated reading comprehension tests and provided meaning for interpreting the difficulty of a book or passage and reading ability of a person (10). The Lexile scale is recognized as the standard for matching readers with texts, being reported for 65 popular reading assessments/programs (24). Over 35 million students per year receive a Lexile measure allowing them to be matched with over 100 million articles books and websites (24).

Stenner and colleagues have long advocated for the application of the methodology used in creating the Lexile to be used in social and behavioral sciences (10, 17). Recently, other research groups have applied a similar methodology to Stenner. These groups include Stenner and Smith (11), Fisher (25), Hong and colleagues (26), Adroher and Tennant (27), and Melin and Pendrill (28–33). In all cases, the research groups created a specification equation using linear, principal component regression, or linear logistic test models composed of component measures to explain the majority of variance accounted for in the observed construct of interest. Constructs of interest were quantified on linear interval scales using Rasch measurement theory models and included visual attention and short term memory with items from the Knox Cube Test (16, 32, 33), physical functioning with items from LSU Health Status Instruments Physical Functioning Scale and SF-36 PF-10 (25, 34), upper extremity function with items from the International Classification of Functioning Activity Measure - Gross Upper Extremity subtest (ICF-GUE) (26), daily activities with items from the Evaluation of Daily Activity Questionnaire (27), non-verbal sequence memory with items from the Corsi Block and Digit Span tests (31), auditory learning with items from the Rey's Auditory Verbal Learning Test (30), and balance with items from the Berg Balance Scale (28). Component measures consisted of continuous or ordinal variables that represent underlying constructs hypothesized to causally contribute to the observed phenomenon. The observed phenomenon was represented and quantified by the item difficulty hierarchies for each construct. The majority of the tested component measures were logically related to task characteristics of the items (16, 25–33). Recently, Melin and Pendrill have also included entropy and measurement uncertainty into their specification equations to account for additional variance (28–33). While each group has been able to account for significant variance (77.5%–94%) (16, 25–33) with their specification equations, which is comparable to Stenner's Lexile work (85%) (10), there have not been any attempts to anchor their specification questions to create universal measurement units.

The body of literature above demonstrates that a theory-based approach can be used to derive mathematical measurement models in the social and behavioral sciences. Furthermore, the Lexile methodology demonstrated the ability to create a universal unit and measurement scale with general objectivity by imposing recognizable anchor points. Application of this methodology to gross motor development should provide a similar quantification of theory and a universal unit for the field. The purpose of this study was to develop a specification equation and a universal measurement unit for gross motor development anchored to well-recognized reference points. We hypothesized that our specification equation would explain significant variation in observable gross motor development.

## Methods

Our methodological approach to develop a specification equation and universal measurement unit for gross motor development included the following steps: (1) selection of gross motor development tasks with observable difficulty levels that are measured on an interval scale, (2) development and scoring of component measures based on hypothesized contributions to gross motor development, (3) testing a specification equation using linear regression modeling, and (4) imposing reference points and unit size by anchoring the specification equation. All analyses were completed in SAS version 9.4.

### Selection of gross motor development tasks with observable difficulty levels

We used the Gross Motor Function Measure-66 (GMFM) as a set of gross motor development tasks with observable difficulty levels. The GMFM is a clinician-observed outcome measure for quantifying child motor development (35). Items were developed with respect to theoretical motor development milestones based heavily on clinical observation of normal child development from 0 to 5 years old (35). Items include tasks that span movements in supine through jumping and hopping. Avery and colleagues (36) reduced the original GMFM from 88 to 66 items by using Rasch measurement theory to identify the set of items that best contributed to unidimensionality of gross motor development. Avery and colleagues (36) used the partial credit model to quantify observed item difficulty along a linear, interval scale using GMFM-66 data from a sample of 537 children with cerebral palsy. We defined gross motor development by increasing difficulty of gross motor tasks. Observed item difficulty for each item on the GMFM-66 was extracted from Avery and colleagues' 2003 analysis (36).

### Development and scoring of component measures for motor development tasks

We identified body position, movement, and support as potential component measures for creating a formula to measure gross motor development. We developed an ordinal rating system for each component based on theoretical concepts of gross motor development and control. Each ordinal rating system was created using task analysis to facilitate reproducibility of component measure scores for raters with knowledge of movement (i.e., task) requirements. Since the tasks on the GMFM-66 are reflective of typical development (35), we selected and rated our component measures based on task characteristics performed by healthy children. Evidence to support our ordinal rating system was based on published observational studies of typical human development and pediatric rehabilitation (35, 37–42). Body position was rated with respect to the theoretical concept that gross motor task difficulty increases as head position and a person's center of mass are further from the ground or are over a smaller base of support (37–39). Movement was rated with respect to established motor development milestones and task difficulty (37, 38, 40). Support was rated based on the amount of support involved in completing the task (35). We quantified support based on the concept of proximal to distal progression of motor control development and considering that more support makes motor tasks easier (41–43). Each member of our authorship team gave each item on the GMFM-66 a rating for one of the three component measures. Three of the authors reviewed the component measure ratings and identified items that did not have consensus for discussion. Items without consensus were discussed and a final decision was made. A full description of the ordinal ratings for each component are presented in Table 1.

**Table 1:** Component measure rating system.

Component Measures					
Rating	Body Position	Rating	Movement	Rating	Support
1	Supine	1	No movement	1	Full proximal and head support
2	Prone	2	Controlled head movement	2	Full proximal support without head support
3	Sitting	3	Rolling	3	Two hand support without proximal support
4	Quadruped	4	Lying to Sitting	4	One hand support without proximal support
5	3-Point	5	Sitting to Quadruped	5	No support
6	Tall Kneed	6	4-Point to 3-Point		
7	Half Kneel	7	Crawling (reciprocal)		
8	Standing	8	Quadruped to Kneel		
9	Standing – Staggered (feet apart)	9	Pull to Stand		
10	Standing – Tandem	10	Sit to Stand		
11	Standing on one foot	11	Stand to Sit		
12	Period without contact with the ground	12	Kneel to Stand		
		13	Cruising		
		14	Walking		
		15	Squatting		
		16	Walking with challenge		
		17	Walking up stairs		
		18	Walking down stairs		
		19	Kicking		
		20	Running		
		21	Jumping		
		22	Hopping on one foot		

Table 1 Presents the rating system for each component measure. Gross motor tasks receive a rating for each component measure. Values for each component measure are used in the specification equation to calculate a measure in Gross Motor (GM) units for the task.

### Testing a specification equation using linear regression modeling

We used linear regression to create a specification equation for measuring gross motor development (10, 16, 25, 26). We used all items on the Gross Motor Function Measure-66 (GMFM) as dependent variables and tested the component measures' (i.e., body position, movement, and support) ability to explain the variance in gross motor task difficulty. Pearson's correlation was used to quantify the linear association between observed task difficulty and each component measure to screen for collinearity and inform a forward stepwise approach for the regression analysis. Collinearity was quantified using the Variance Inflation Factor (VIF). We considered VIF greater than or equal to 10 as the threshold for determining whether two variables were collinear (44). If collinearity was found, one of the two colinear variables would be removed after evaluating each variable for statistical significance and theoretical implications. Forward stepwise linear regression was used to quantify the amount of variance in observed task difficulty of items on the GMFM-66 using component measures for body position, movement, and support for each item. The final regression model was used to calculate predicted difficulty for each GMFM-66 item based on component measures. We used Pearson's correlation to quantify the agreement between observed and predicted task difficulty of each item.

### Measurement unit and scale anchoring with reference points

We used the final regression model as a specification equation to define the gross motor development measurement scale and finalize a formula for measuring the difficulty of any gross motor task. We imposed reference points on the measurement scale by identifying two anchor points. We selected the item “supine brings hands to midline” as the low anchor and “walking with hands free” as the high anchor. The specification equation was used to calculate the predicted task difficulty measures for the low and high anchors. These predicted task difficulty measures were used to algebraically anchor the specification equation to the points 0 (“supine brings hands to midline”) and 100 (“walking with hands free”) establishing a unit size equal to 1/100 of the distance between lying supine and walking unsupported.

## Results

**Figure 1:**
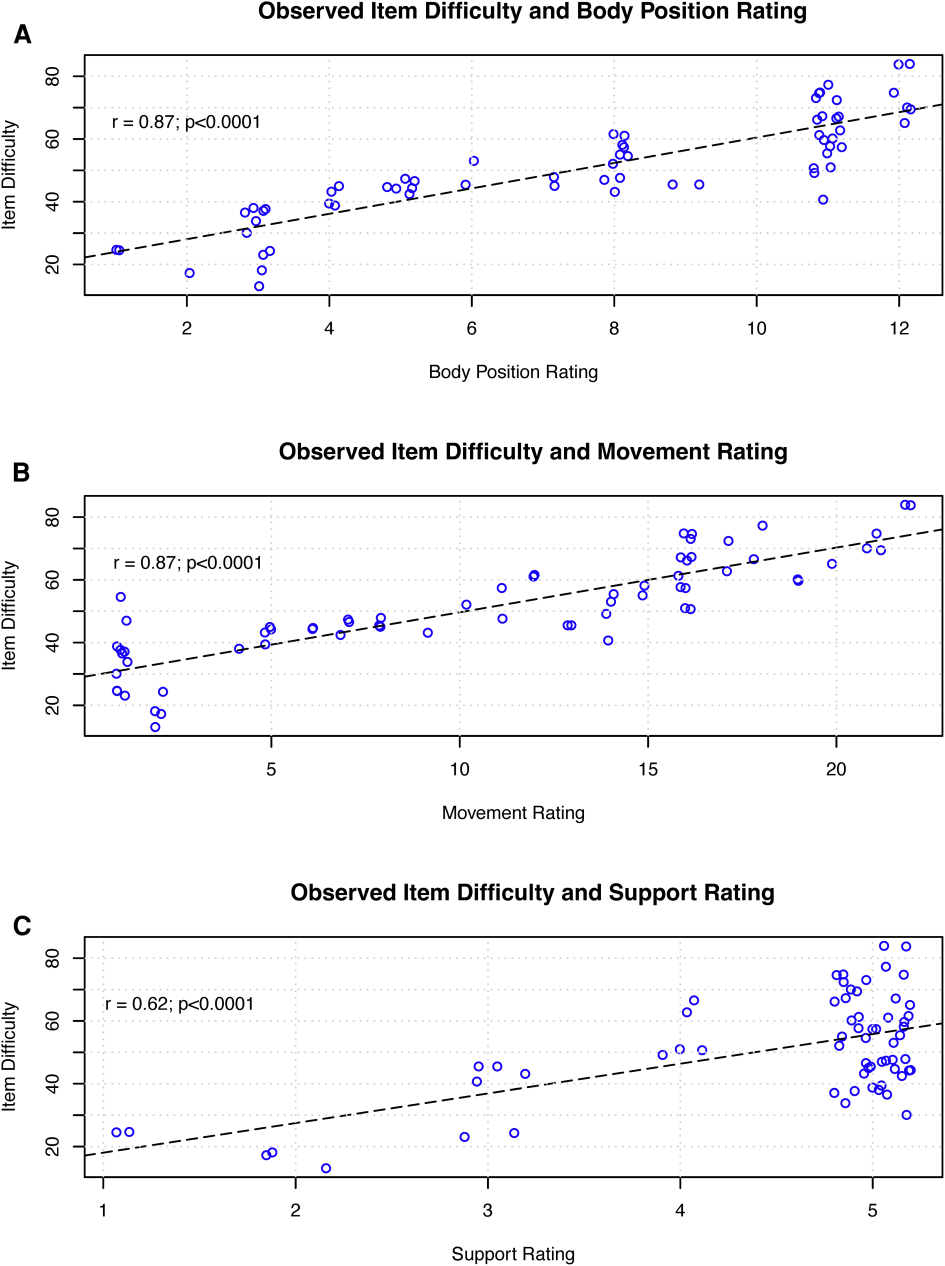
Figure 1 presents the linear association between observed task difficulty and each component measure for each item on the GMFM. Each circle represents an individual item on the GMFM. The dashed line represents the best fit linear model. (**A**) presents the relationship for body position rating, (**B**) for movement rating, and (**C**) for support rating.

Table 2 presents the component measure ratings for all items on the GMFM. All component measures have a positive, significant linear association with observed rank difficulty. Figure 1 presents scatterplots for the ratings of each component measure, body position (Figure 1A), movement (Figure 1B) and support (Figure 1C), against the item difficulty for each item on the GMFM. All correlations were significant (*p* < .001); body position and movement correlated with item difficulty at 0.87 and support ratings correlated at 0.62) (Figure 1). A linear regression model with all three component measures explained the most variance in GMFS item observed rank order difficulty (adjusted *R*^2 ^= 0.87; *F*-value = 147.01, *p* < 0.0001, RMSE = 6.09). None of the VIF values exceeded our threshold of collinearity. The estimated effect and standard error of each component measure on gross motor task difficulty were body position; *β* = 1.23, SE = 0.59 (*p* = 0.0415), movement; *β* = 1.21, SE, 0.30 (*p* = 0.001), support; *β* = 4.93, SE = 0.74 (*p* < 0.0001).

**Table 2:** Component measure ratings for each item on the GMFM.

Gross Motor Function Scale Items		Component Measures		
Item Number	Item Name	Body Position	Movement	Support
2	SUP: BRINGS HANDS TO MIDLINE	1	1	1
6	SUP: R HAND CROSSES MIDLINE	1	1	1
7	SUP: L HAND CROSSES MIDLINE	1	1	1
10	PR: LIFTS HEAD UPRIGHT	2	2	2
18	SUP: PULLS TO SITTING	3	2	3
21	SIT: LIFTS HEAD UPRIGHT	3	2	2
22	LIFTS HEAD TO MIDLINE	3	2	2
23	SIT: ARMS PROPPING	3	1	3
24	SIT: ARMS FREE	3	1	5
25	SIT: WITH TOY	3	1	5
26	SIT: REACH BEHIND TO R	3	1	5
27	SIT: REACH BEHIND TO L	3	1	5
30	SIT: LOWERS TO PRONE	3	4	5
31	SIT ON MAT: TO 4PT OVER A SIDE	5	5	5
32	SIT ON MAT: TO 4PT OVER L SIDE	4	5	5
34	SIT: ARMS & FEET FREE	3	1	5
35	STD: ATTAINS SIT	8	11	5
36	ON FLOOR: ATTAINS SIT SMALL BENCH	7	8	5
37	ON FLOOR: ATTAINS SIT LARGE BENCH	7	8	5
39	4-POINT: MAINTAINS 4PT	4	1	5
40	4-POINT: ATTAINS SIT	4	5	5
41	PRN: ATTAINS 4PT	4	5	5
42	4-POINT: REACHES WITH R	5	6	5
43	4-POINT: REACHES WITH L	5	6	5
44	4-POINT: CRAWLS FORWARD 1.6 m	5	7	5
45	4-POINT: CRAWLS RECPRCLLY 1.0 m	5	7	5
46	4-POINT: CRAWL UP 4 STEPS	5	7	5
48	SIT ON MAT: ATTAIN HIGH KN ARMS FREE	6	8	5
51	HIGH KN: KN WALK FORWARD	6	14	5
52	ON FLOOR: PULL TO STAND AT BENCH	8	9	3
53	STD: ARMS FREE, 3 SEC	8	1	5
54	STD: LIFT R FOOT	11	16	4
55	STD, LIFT L FOOT	11	16	4
56	STD: ARMS FREE 10 SEC	8	1	5
57	STD: LIFT LFT ARMS FREE	11	16	5
58	STD: LIFT RFT ARMS FREE	11	16	5
59	SIT BENCH: ATTAINS STD ARMS FREE	8	10	5
60	HIGH KN: TO STAND A SIDE	8	12	5
61	HIGH KN: TO STAND L SIDE	8	12	5
62	STD: LOWERS TO SIT, ARMS FREE	8	11	5
63	STD: TO SQUAT, ARMS FREE	8	15	5
64	STD: PICK UP OBJ ARMS FREE	8	15	5
65	STD: CRUISE TO R WITH BENCH	9	13	3
66	STD: CRUISE TO L WITH BENCH	9	13	3
67	STD: WALK WITH HANDS HELD	11	14	3
68	STD 1 HAND HELD: WALK WITH 1 HAND HELD	11	14	4
69	STD: WALK, HANDS FREE	11	14	5
70	STD: WALK 10 STEPS, RETURN	11	16	5
71	STD: WALK BACKWARD 10 STEPS	11	16	5
72	STD: WALK WITH OBJECT HELD	11	16	5
73	STD: WALK BETWEEN LINES	11	16	5
74	STD: WALK ALONG STRAIGHT LINE	11	16	5
75	STD: STEP OVER STICK, A FOOT	11	16	5
76	STD: STEP OVER STICK, L FOOT	11	16	5
77	STD: RUN 4.5 m, RETURN	12	20	5
78	STD: KICK BALL, R FOOT	11	19	5
79	STD: KICK BALL, L FOOT	11	19	5
80	STD: JUMP UP 30 cm BOTH FEET	12	21	5
81	STD: JUMP FWD 30 cm BOTH FEET	12	21	5
82	STD L FOOT: HOP 10X, R FOOT	12	22	5
83	STD L FOOT: HOP 10X, L FOOT	12	22	5
84	STD, HOLDING 1 RAIL: UP 4 STEPS	11	17	4
85	STD: DOWN 4 STEPS HLDING RAIL	11	18	4
86	STD: UP 4 STEPS, NO RAIL	11	17	5
87	STD: DOWN 4 STEPS, NO RAIL Difficult	11	18	5
88	STD 6” STEP: JUMP OFF 15 cm STP 2 FEET	12	21	5

Table 2 Presents the consensus component measure ratings for each item on the GMFM. Items are listed in order of item number on the GMFS with their short name reported in Avery et al., 2003.

The final specification equation is:

TaskDifficulty=6.76+1.23∗(BodyPosition)+1.21∗(Movement)+4.93∗(Support)

**Figure 2:**
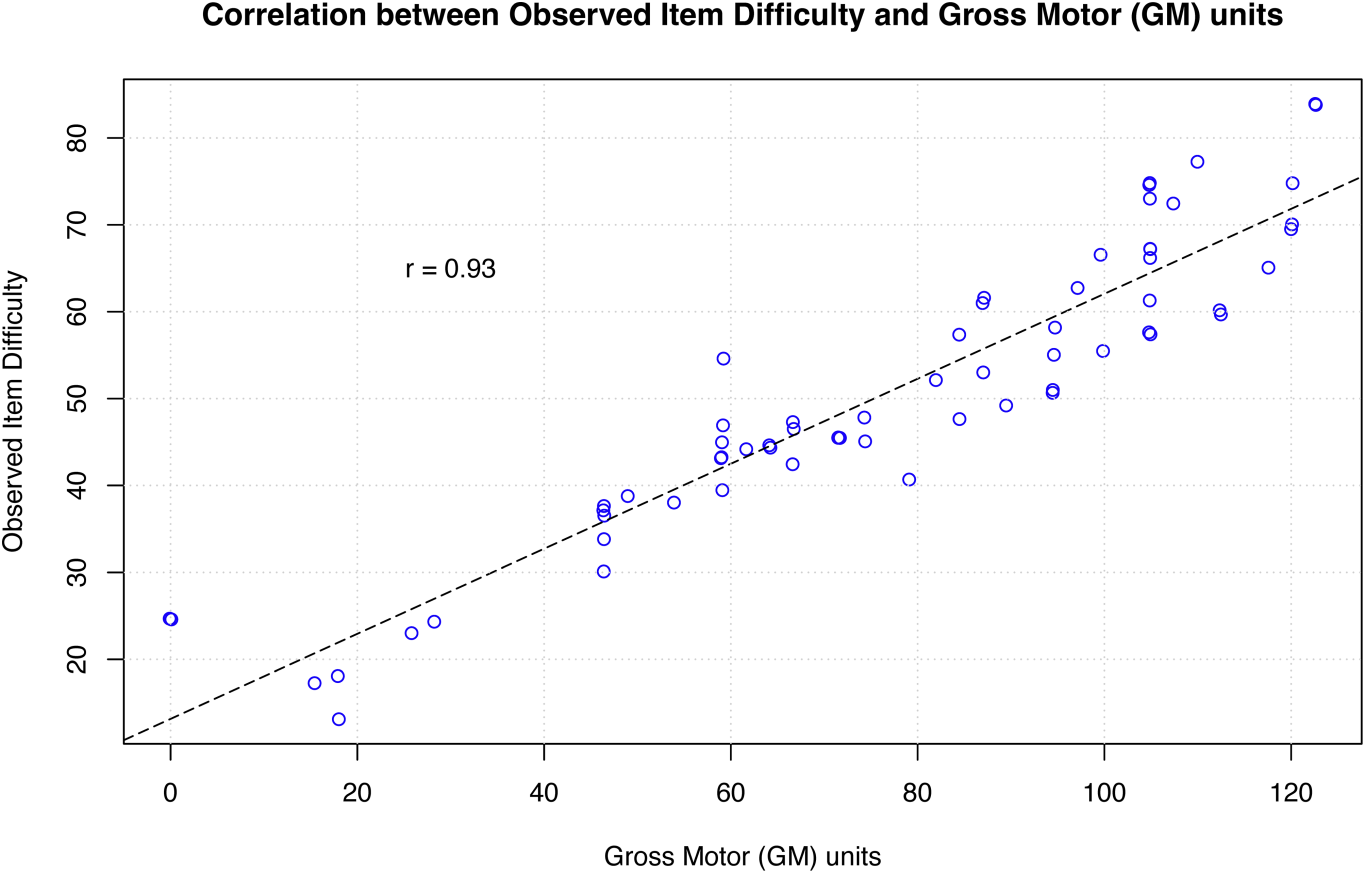
Figure 2 Presents the linear association between observed task difficulty and GM units for each item on the GMFM. Each circle represents an individual item on the GMFM. The dashed line represents a line of equality (or identity line) where *r* = 1.0. The correlation between observed task difficulty and GM units is *r* = 0.93.

Predicted task difficulty has a positive, significant linear association with observed rank difficulty (*r* = 0.93 *p* < 0.001). Figure 2 presents a scatterplot of predicted task difficulty against observed task difficulty for each item on the GMFM.

### Gross motor development measurement scale and unit

The predicted task difficulty for our low anchor (“supine brings hands to midline”) was 14.13. We assigned a value of 0 to the low anchor. We used the following equation to derive a constant that can be used to anchor the specification's predicted task difficulty of “supine brings hands to midline” to 0:

$$0+(\mathrm{constan}t_{\mathrm{low}\;\mathrm{anchor}}+14.13)=\;0\;\mathrm{Gross}\;\mathrm{Motor}\;(\mathrm{GM})\;\mathrm{units}$$

where

$$\mathrm{constan}t_{\mathrm{low}\;\mathrm{anchor}}=(-14.13)$$

The predicted task difficulty for our high anchor (“walking with hands free”) was 61.88. We assigned a value of 100 to the high anchor to set our unit size to 1/100. We used the following equation to derive a constant that can be used to anchor the specification's predicted task difficulty of “walking with hands free” to 100:

$$\mathrm{constan}t_{\mathrm{high}\;\mathrm{anchor}}\;(\mathrm{constan}t_{\mathrm{low}\;\mathrm{anchor}}+61.88)=\;100\;\mathrm{Gross}\;\mathrm{Motor}\;(GM)\;units$$

$$\mathrm{constan}t_{\mathrm{high}\;\mathrm{anchor}}\;(-14.13\;+\;61.88)\;\;=\;100\;\mathrm{Gross}\;\mathrm{Motor}\;(\mathrm{GM})\;\mathrm{units}$$

$$\mathrm{constan}t_{\mathrm{high}\;\mathrm{anchor}}\;(47.75)=100$$

$${\mathrm{constant}}_{\mathrm{high}\;\mathrm{anchor}}=100/(47.75)$$

$${\mathrm{constant}}_{\mathrm{high}\;\mathrm{anchor}}=2.09$$

The final equation that places predicted (i.e., theoretical) gross motor task difficulty on the measurement scale in Gross Motor units can be written using the high and low anchor constants as follows:

(TaskDifficulty−14.13)∗2.09=GrossMotor(GM)units

where task difficulty is the predicted task difficulty from the specification equation. Table 3 presents the GM units for each item on the GMFM.

**Table 3:** Gross motor (GM) units for each item on the GMFM.

Item Number	Item Name	Observed Difficulty	Gross Motor (GM) units
*2	SUP: BRINGS HANDS TO MIDLINE	14.13	0
6	SUP: R HAND CROSSES MIDLINE	14.13	0
7	SUP: L HAND CROSSES MIDLINE	14.13	0
10	PR: LIFTS HEAD UPRIGHT	21.5	15
22	LIFTS HEAD TO MIDLINE	22.73	18
21	SIT: LIFTS HEAD UPRIGHT	22.73	18
23	SIT: ARMS PROPPING	26.45	26
18	SUP: PULLS TO SITTING	27.66	28
24	SIT: ARMS FREE	36.31	46
25	SIT: WITH TOY	36.31	46
34	SIT: ARMS & FEET FREE	36.31	46
27	SIT: REACH BEHIND TO L	36.31	46
26	SIT: REACH BEHIND TO R	36.31	46
39	4-POINT: MAINTAINS 4PT	37.54	49
30	SIT: LOWERS TO PRONE	39.94	54
52	ON FLOOR: PULL TO STAND AT BENCH	42.28	59
41	PRN: ATTAINS 4PT	42.38	59
40	4-POINT: ATTAINS SIT	42.38	59
32	SIT ON MAT: TO 4PT OVER L SIDE	42.38	59
53	STD: ARMS FREE, 3 SEC	42.46	59
56	STD: ARMS FREE 10 SEC	42.46	59
31	SIT ON MAT: TO 4PT OVER A SIDE	43.61	62
43	4-POINT: REACHES WITH L	44.82	64
42	4-POINT: REACHES WITH R	44.82	64
44	4-POINT: CRAWLS FORWARD 1.6m	46.03	67
45	4-POINT: CRAWLS RECPRCLLY 1.0m	46.03	67
46	4-POINT: CRAWL UP 4 STEPS	46.03	67
65	STD: CRUISE TO R WITH BENCH	48.35	72
66	STD: CRUISE TO L WITH BENCH	48.35	72
48	SIT ON MAT: ATTAIN HIGH KN ARMS FREE	48.47	72
36	ON FLOOR: ATTAINS SIT SMALL BENCH	49.7	74
37	ON FLOOR: ATTAINS SIT LARGE BENCH	49.7	74
67	STD: WALK WITH HANDS HELD	52.02	79
59	SIT BENCH: ATTAINS STD ARMS FREE	53.35	82
35	STD: ATTAINS SIT	54.56	84
62	STD: LOWERS TO SIT, ARMS FREE	54.56	84
51	HIGH KN: KN WALK FORWARD	55.73	87
61	HIGH KN: TO STAND L SIDE	55.77	87
60	HIGH KN: TO STAND A SIDE	55.77	87
68	STD 1 HAND HELD: WALK WITH 1 HAND HELD	56.95	89
54	STD: LIFT R FOOT	59.37	95
55	STD, LIFT L FOOT	59.37	95
63	STD: TO SQUAT, ARMS FREE	59.4	95
64	STD: PICK UP OBJ ARMS FREE	59.4	95
84	STD, HOLDING 1 RAIL: UP 4 STEPS	60.58	97
85	STD: DOWN 4 STEPS HLDING RAIL	61.79	100
* 69	STD: WALK, HANDS FREE	61.88	100
70	STD: WALK 10 STEPS, RETURN	64.3	105
72	STD: WALK WITH OBJECT HELD	64.3	105
57	STD: LIFT LFT ARMS FREE	64.3	105
58	STD: LIFT RFT ARMS FREE	64.3	105
71	STD: WALK BACKWARD 10 STEPS	64.3	105
73	STD: WALK BETWEEN LINES	64.3	105
75	STD: STEP OVER STICK, A FOOT	64.3	105
76	STD: STEP OVER STICK, L FOOT	64.3	105
74	STD: WALK ALONG STRAIGHT LINE	64.3	105
86	STD: UP 4 STEPS, NO RAIL	65.51	107
87	STD: DOWN 4 STEPS, NO RAIL Difficult	66.72	110
78	STD: KICK BALL, R FOOT	67.93	112
79	STD: KICK BALL, L FOOT	67.93	112
77	STD: RUN 4.5m, RETURN	70.37	118
80	STD: JUMP UP 30cm BOTH FEET	71.58	120
81	STD: JUMP FWD 30cm BOTH FEET	71.58	120
88	STD 6" STEP: JUMP OFF 15cm STP 2 FEET	71.58	120
82	STD L FOOT: HOP 10X, R FOOT	72.79	123
83	STD L FOOT: HOP 10X, L FOOT	72.79	123

Table 3 presents the observed task difficulty and measure in Gross Motor (GM) units for each item on the GMFM. Items are ordered based on GM units. Less GM units indicate easier task difficulty and more GM units indicate harder task difficulty. Reference points for anchors are denoted with *. GM units are derived from predicted task difficulty calculated by the specification equation based on theory.

## Discussion

We demonstrated that component measures of body position, movement, and support during gross motor tasks can explain the majority of variance in gross motor development (adjusted *R*^2^ = 0.87) using linear regression. The findings from our analysis were concordant with previously published results where linear regression models were used; Stenner (10), Stenner and Smith (16), Fisher (25), and Hong and colleagues (26). Our explanation of 87% of the variance of our model was similar to the 85% of variance explained by the Lexile linear regression model (10) and the 83% of the variance explained by the ICF-GUE linear regression model (26). The linear regression for the Knox Cube Test (16) and physical function (25) explained considerably more variance, 95% (16) and 94% (25). This may suggest that there is a better understanding of, or ability to quantify, the components accounting for task difficulty in visual attention and short-term memory, and physical function compared to our understanding of gross motor task difficulty. While linear regression is a popular method for deriving specification equations, there are groups testing other methods such as a linear logisitic model, partial least squares model, and principal component regression. Work by Adroher and Tennant (27), Pendrill (32), and Melin and colleagues (28–31, 33) have used these models to address limitations of linear regression when using ordinal variables or to account for more complex variables such as measurement uncertainty or entropy.

Our ability to establish a universal unit for gross motor development applied the same methodology used by Stenner (10) to create the Lexile for reading. We anchored our equation and measurement scale to lying supine (0 GM units, low level) and walking (100 GM units, high level) because, like basal level and encyclopedia level texts, these tasks are widely recognized and easily reproducible (37, 40–42). Lying supine is widely recognized as the first developmental task a child can do after birth while walking is a critical childhood developmental milestone and universal characteristic of the human experience. We created a 100-point gross motor development scale since 100 point scales are commonly understood and easily communicated between healthcare providers and patient or patient families (12). It is important to recognize that measurement scales extend to infinity in both directions despite anchoring. For example, temperature can still be measured on the Celsius scale below 0° and above 100°. The gross motor development scale begins at lying supine (which we identified as the lowest level of gross motor development) but extends beyond walking to more difficult gross motor tasks. This is apparent on our measurement scale with tasks like jumping and stair navigation receiving measures of 110 and 120 GM units, respectively.

The process of imposing anchor points and unit size to create universal units also provides frames of reference for interpretation of measures (11, 32). Currently, interpretation of measurement values from existing instruments typically requires normative data and large group comparisons. Anchored measurement scales to well-known references and universal units removes this barrier. For example, Lexile measures are interpreted with respect to the anchor texts and Lexile unit size; “a passage with a Lexile of 1,000 is much more difficult than a text with a Lexile of 600”, or “a passage with a Lexile of 800 is 600 Lexiles away from the difficulty of a basal reading text and 400 Lexiles away from the difficulty of an encyclopedia passage”. Additionally, Lexiles have an interval, fixed unit size which means a change from 200 to 400 is the same “distance” and comparable to a change from 600 to 800. The gross motor development measurement scale is also linear and interval which allows for the same interpretation of GM units. For example, a child who can sit unsupported (46 GM units) is 54 GM units away from walking and a child who can walk with handheld support (79 GM units) is 21 GM units away from walking unsupported. This is also similar to the way a ruler is used to measure length and then compare the length of two objects or a change in an object's length. Theoretically, the Gross Motor unit also has a degree of general objectivity since the unit size and measurement scale is not derived from a specific sample (local objectivity) but rather the theoretical model (specification equation). This should allow GM units to be interpreted the same regardless of whether a child is healthy or has a condition that results in developmental delays. Future research is needed to confirm this capability.

The theory behind the construct of gross motor development is a narrative about how humans gain the ability to do more difficult tasks based on evidence and observation. Our work demonstrates that movement along the developmental progression can be mathematically modeled using a regression formula informed by theory that accurately predicts the gross motor task difficulty hierarchy. The extent to which the formula can accurately predict informs the theory's adequacy by testing and exposing the theory to falsification (10, 16). We tested aspects of gross motor development theory by including body position, movement, and support component measures in our specification equation. Our equation's ability to accurately predict developmental progression demonstrates that several theoretical aspects of the gross motor development narrative and construct hold up to falsification. This methodology to measure gross motor development centers on elucidating the relationships between item characteristics and gross motor task difficulty as this provides a more thorough understanding of what critical components underly gross motor development and it's variations (32).

### Implications and future research

Creation of specification equations holds three key benefits for social and behavioral research (16, 11). First, it states theory in a way that makes falsification possible. Second, component measures in a specification equation can often be measured with more precision ultimately reducing error as equations are refined (16). Third, experimental manipulation is feasible because items or tasks can be derived from the specification equation and their difficulty can be tested (19). It is important to remember that as theories are exposed to falsification, specification equations can be improved through the refinement or removal of component factors and with the inclusion of new ones as greater understanding of constructs are uncovered. Furthermore, exposing constructs to falsification is the “challenge research” that should focus and accelerate future theory-based discovery (45).

The implications of creating a universal measurement unit and scale in the social and behavioral sciences using theoretical considerations independent of sampling are far reaching. First, and most important, is that a specification equation provides a means to calibrate most, if not all, items measuring a construct onto the same scale. Future research will need to test and validate our specification equation against findings from 1) other regression modeling methods, 2) other populations and 3) independent item sets like the Peabody Developmental Motor Scales and Denver Developmental Screening Test. This would establish whether all gross motor development items can be placed onto the same measurement scale in GM units. This should allow for most, if not all, existing gross motor development instruments to be anchored on a single measurement scale with linear, interval units derived from theory. Second is the ability to facilitate efficient measurement for children's gross motor development. Children can be given a GM unit measure based on logically selecting a few tasks to see if the child can accomplish the tasks or observing the child completing tasks in a natural play setting. Additionally, we no longer need to be concerned with item banks or completing all items on an instrument because any gross motor task can be created and rated using the component measures and the specification equation. Furthermore, additional component measures should be explored like measurement uncertainty, entropy, other hypothesized contributing factors to development to refine our understanding and ability to measure gross motor development (32, 33). Lastly, future research should aim to identify objective or continuous variables for component measures.

### Limitations

Our study has several limitations. First, our regression analysis and specification equation was derived from observed item difficulty for children with cerebral palsy (36). Future studies should observe item difficulty in a sample of typically developing children to determine if disease condition influences the regression findings. Second, we did not compare our results from linear regression to those from other regression models. Although linear regression can accommodate ordinal independent variables, the beta estimates cannot be interpreted on an interval scale (46). Researchers should be aware of this limitation when predicting changes in gross motor task difficulty from beta estimates for component measures. Third, our specification equation accounted for 87% of the variance, which indicates that there is still 13% variance unexplained. While no equation can account for 100% of the variance due to error, additional components of gross motor development theory can be tested which may improve the accuracy of the specification equation. Fourth, we did not account for measurement uncertainties or entropy in our specification equation. This is an emerging methodology and may allow for deeper understanding of constructs in social and behavioral sciences (28–33) Fifth, future research is needed to evaluate our measurement theory's ability to hold up to falsification. This future research should further our understanding of gross motor development and improve the accuracy and precision of our measurement specification equations.

## Conclusion

We have demonstrated that the methodology to develop anchored specification equations, like the Lexile measurement scale, can be applied to gross motor development. We have shown that a specification equation for gross motor development can account for the majority of variance in task difficulty. Additionally, we showed that anchoring specifications algebraically can achieve general objectivity to create a universal unit of gross motor measurement (i.e., GM unit). Equipped with a measurement equation and universal unit of measurement, most, if not all, existing gross motor development instruments should be able to be calibrated to the same scale with a linear, interval fixed unit.

## Data Availability

The original contributions presented in the study are included in the article/supplementary material, further inquiries can be directed to the corresponding author.
